# Phylotyping and Functional Analysis of Two Ancient Human Microbiomes

**DOI:** 10.1371/journal.pone.0003703

**Published:** 2008-11-11

**Authors:** Raúl Y. Tito, Simone Macmil, Graham Wiley, Fares Najar, Lauren Cleeland, Chunmei Qu, Ping Wang, Frederic Romagne, Sylvain Leonard, Agustín Jiménez Ruiz, Karl Reinhard, Bruce A. Roe, Cecil M. Lewis

**Affiliations:** 1 Department of Anthropology, University of Oklahoma, Norman, Oklahoma, United States of America; 2 Molecular Anthropology Laboratories, University of Oklahoma, Norman, Oklahoma, United States of America; 3 Department of Chemistry and Biochemistry, Advanced Center for Genome Technology, University of Oklahoma, Norman, Oklahoma, United States of America; 4 Institute of Technology, University of Auvergne, Auvergne, Clermont-Ferrand, France; 5 University of Nebraska State Museum, Lincoln, Nebraska, United States of America; 6 School of Natural Resources, University of Nebraska, Lincoln, Nebraska, United States of America; Centre for DNA Fingerprinting and Diagnostics, India

## Abstract

**Background:**

The Human Microbiome Project (HMP) is one of the U.S. National Institutes of Health Roadmap for Medical Research. Primary interests of the HMP include the distinctiveness of different gut microbiomes, the factors influencing microbiome diversity, and the functional redundancies of the members of human microbiotas. In this present work, we contribute to these interests by characterizing two extinct human microbiotas.

**Methodology/Principal Findings:**

We examine two paleofecal samples originating from cave deposits in Durango Mexico and dating to approximately 1300 years ago. Contamination control is a serious issue in ancient DNA research; we use a novel approach to control contamination. After we determined that each sample originated from a different human, we generated 45 thousand shotgun DNA sequencing reads. The phylotyping and functional analysis of these reads reveals a signature consistent with the modern gut ecology. Interestingly, inter-individual variability for phenotypes but not functional pathways was observed. The two ancient samples have more similar functional profiles to each other than to a recently published profile for modern humans. This similarity could not be explained by a chance sampling of the databases.

**Conclusions/Significance:**

We conduct a phylotyping and functional analysis of ancient human microbiomes, while providing novel methods to control for DNA contamination and novel hypotheses about past microbiome biogeography. We postulate that natural selection has more of an influence on microbiome functional profiles than it does on the species represented in the microbial ecology. We propose that human microbiomes were more geographically structured during pre-Columbian times than today.

## Introduction

Humans are adapted superorganisms harboring as many as 1000 different microbial species within our intestine that collectively constitute our microbiome. The microbiome co-evolves with the host [1] and provides the ability to harvest nutrients and produce additional energy that are otherwise inaccessible to the host. These microbes produce vitamins, metabolize xenobiotics, provide resistance to tumor and cancer leading neoplasms, and assist in developing a mature immune system [2]. Not surprisingly, human microbiotas are the focus of the NIH Human Microbiome Project [1], [2] and are included in a wide range of other health and disease studies from peptic ulcers [3], kidney stones [4], neurological phenotypes [5], [6], cancer [2], cardiovascular disease [7], and obesity and diabetes [8]–[14]. In addition to influencing human disease, microbiotas can influence treatments for disease through drug metabolism [15] leading to speculation that these ecosystems may result in an untapped source of novel drug treatments [16]. However, at present, little is known about how distinct these intestinal microbes are, what influences their diversity and how functionally redundant are its members [2], [9].

Our microbiome provides biological adaptations that we did not evolve on our own. In previous studies, the evolutionary processes of microbiomes could only be inferred from comparative analysis of extant mammals [1]. We show here that we can interrogate extinct microbiomes by paleogenomics to understand past microevolutionary processes.

Ancient human microbiome studies provide a view of these ecologies prior to global immigration, industrialization, and modern medicine. The human socioeconomic environment has changed dramatically. For example, the abundant use of antibiotics in food preparation and medical practice has disrupted our immune systems ability to target pathogens while avoiding mutualistic bacteria [17]. It is clear that antibiotics taken in the early years of life considerably increase the risk of asthma [18], the “hygiene hypothesis” [19], that a more hygienic environment results in an imbalanced T-cell development, has been postulated as a potential explanation.

**Figure 1:**
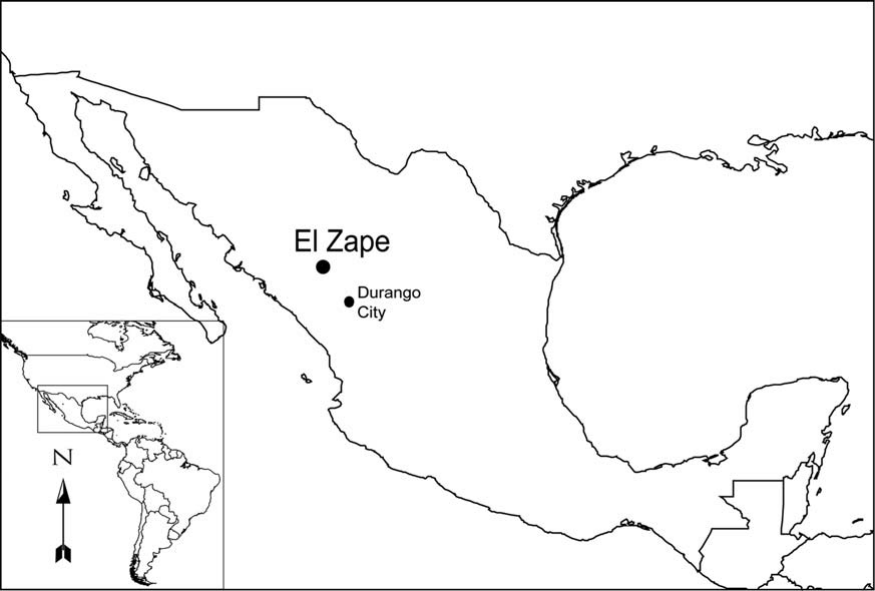
Location of El Zape with respect to Durango City, Mexico.

To study microbiomes prior to the modern condition, we have analyzed the phylotypes and characterized the metabolic potential of two ancient human microbiomes. Additionally, we have developed effective methods to control for DNA contamination during shotgun sequencing by ligating multiplex identifiers (MIDs) to the ancient DNA extracts prior to the reagents leaving a controlled laboratory environment. These ancient DNA samples originated from two human coprolites (paleofeces) from midden deposits from the Cueva de los Muertos Chiquitos archaeological site near Rio Zape, Durango, Mexico (Figure 1). We refer to the samples as Z1 and Z2, respectively. Analysis of diagnostic markers for Native American mitochondrial haplogroups revealed that each coprolite originated from a different person, belonging to haplogroups B and D, respectively. Wood samples associated with the Zape coprolites radiocarbon date to 1300±100 years ago, consistent with the Loma San Gabriel culture. Raised 50 feet above the Rio Zape and accessible by hand and toe holds [20], the cave site has reduced access by animals and thereby provides a unique opportunity to study coprolites that were undisturbed.

## Results and Discussion

Controlling for DNA contamination during pyrosequencing was a primary concern as this approach differs from conventional PCR-based Sanger sequencing. After extracting the coprolite DNA, shotgun sequencing data was obtained using a 454/Roche GS FLX pyrosequencing system. Emulsion PCR steps are required in the 454 shotgun sequencing protocol. This provides an increased risk for modern contamination because these steps are performed outside of a strict contamination controlled ancient DNA clean room. To detect DNA contamination prior to emulsion PCR, we tagged each sample with a unique MID while the samples were in the dedicated ancient DNA clean room. In an ideal situation, where the 454/Roche GS-FLX (FLX) run is of perfect quality, and no contamination was present, each read would sequence one of two MIDs. A deviation from this pattern assesses contamination and run quality. Our FLX run yielded 30,705 and 14,118 reads for sample Z1 and Z2, respectively. We observed two reads with MIDs not used in this study and 430 reads that lacked any recognizable MID, which could reflect contamination that occurred sometime after sample ligation but more likely indicates reads of too poor quality to identify the MID. The number of reads for samples Z1 and Z2 is consistent with that expected given the size of the FLX run.

**Figure 2:**
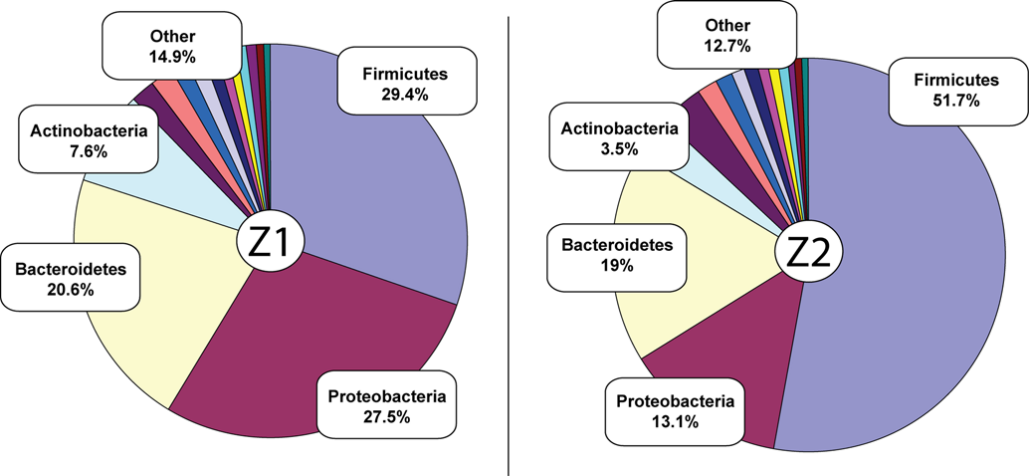
Frequencies of phyla represented in the ancient gut samples. Only divisions comprising greater than 0.5% of the reads are shown. Details on “Other” divisions are provided on Table S1.

The modern microbial diversity of the mammalian gastrointestinal track, with as many as 1000 different species, can be described as diverse but specialized with typically fewer than nine bacterial phyla [21], compared to the over 20 bacterial phyla observed in soil [22]. Furthermore, much of the mammal gut is dominated by Bacteroidetes, Firmicutes, Actinobacteria and Proteobacteria [1], [9], [23], [24] with their respective frequencies associated with the dietary practices of the species [1] and with disease predisposition [2], [9], [25]. We matched 34,585 reads successively using BLASTN on the whole NCBI refseq_genomic database, 26,788 and 7,797 reads for samples Z1 and Z2, respectively. When the BLASTN results were screened for matches with identity greater than 80%, 5115 reads for Z1 and 2057 reads for Z2 qualified, reflecting 16% of the total data. Table S1 provides the count and percentages of the divisions matched. The dominance of Bacteroidetes, Firmicutes, Actinobacteria and Proteobacteria in both samples validates these data as reflecting a gut/fecal ecology. The frequencies of Firmicutes and Proteobacteria varied greatly between the two ancient samples (Figure 2) as has been observed in modern human studies [24] with individual differences attributed to factors such as diet, sex, and/or disease [2], [9], [25].

In the phylotyping of ancient DNA shotgun reads, several concerns must be addressed. Our matches represent sequences that are random areas of the genome; they are unlikely to be as robust as matches derived from hypervariable loci, such as the 16S ribosomal DNA [25]. False matches during shotgun sequencing are a concern [26]. Fortunately, our shotgun phylotyping results were consistent with a human microbiome profile, which was unlikely to be attributed to chance. Importantly, 16S ribosomal studies of ancient DNA have other challenges. Ancient DNA molecules are typically between 50–500bp [27]. Consequently, custom ancient DNA primers could flank only a small area of the 16S locus. To date, it is uncertain how universal or representative such custom primers would be of bacterial species [28].

When we examined the phylotype diversity below the level of division by screening the BLASTN results under stricter criteria, 70% read coverage and 90% identity, all the BLASTN matches had “Total Scores” greater than 100. The species with the highest Max Scores were recorded. 422 reads, approximately 1% of the total sample, fit these criteria. At the level of genus and species, several of the reads with high identities were of special interest as they reflect pathogenic organisms. Further considerations of these matches are required.

*Neisseria* emerged as the best match based on Max Score in several matches. Sixteen of the reads in the ancient samples matched the *Neisseria* genus with greater than 80% identity. Although one read had a 94 percent identity match to *Neisseria gonorrhoeae,* it was not exclusive to the *Neisseria* genus; various “uncultured” bacteria had similar levels of identity and coverage. Because the database was limited to two species of *Neisseria*, *Neisseria gonorrhoeae* and *Neisseria meningitides*, it is possible that the reads represent commensal organisms within the Neisseriaceae family or *Neisseria* genus.

Eighteen reads matched *Yersinia* species. Three reads had greater than 90 percent identity to *Yersinia enterocolitica.* No other genera of Enterobacteriaceae matched these criteria.

Several reads matched both commensal and pathogenic organisms with equally strong identity percentages. Of those reads with greater than 80% identity, 34 matched the *Plasmodium* genera, 14 matched *Shigella* genera, 12 matched *Mycobacterium tuberculosis* complex, and 11 matched *Leishmania*. These genera will be targets for future study.

Approximately 1.7 percent of the sequences had homology to plant or animal DNAs and one percent was homologous with fungi. The major species represented were *Zea mays* (maize), *Homo sapiens*, and the fungal plant pathogens *Eremothecium gossypii* and *Gibberella zeae*. The trace number of human sequences observed may represent a small number of host cells present in the microbiome or contamination prior to MID ligation. The presence of maize DNA is expected because maize was an important subsistence resource in this region of Mexico and was present within the archaeological site. Interestingly, the division Archaea was represented exclusively by *Methanobrevibacter smithii*, which is the only species of Archaea known to be common in the human gut.

A typical microbial ecology often contains species that are functionally redundant [29]. Although many of these species will be in lower frequency compared to the dominate species of the system, the accumulation of the lower frequency species can quickly result in a dominant functional influence. An alternative, genecentric, study focuses on the functional aspects of the microbiome by profiling orthologous genes rather than determining the species from which they derived. This is an approach to fingerprint environments [29] that may be extended to human conditions.

Current research begins to define the variation in the functional aspects of human microbiomes revealing an enrichment of several pathways including carbohydrate and amino acid metabolism [23] when compared to the Kyoto Encyclopedia of Genes and Genomes (KEGG) and Clusters of Orthologous Groups (COG) databases [29]. Since KEGG pathway data for modern human samples were available [23], we compared them to our ancient data. When samples Z1 and Z2 were examined together, 6978 and 8490 putative open reading frames were matched to KEGG (Table S2) and COG (Table S3) databases, respectively. As expected, the pathways involved in carbohydrate and amino acid metabolism were enriched (Table 1). Surprisingly, the frequencies of KEGG and COG pathways between the two ancient samples were very similar, particularly when compared to modern data. While this can be explained by differences in analytical methods between the modern and ancient study, the level of similarity between the ancient gut samples is striking. This similarity cannot be attributed to random sampling of the KEGG and COG databases (Table 2).

**Table 1:** KEGG pathway data comparing modern and ancient fecal samples.

	Modern*		Ancient			
KEGG Category Pathway	Hits	Percent	Z1 Hits	Z2 Hits	Z1+Z2 Hits	Z1+Z2 Percent
Carbohydrate Metabolism	5473	53.57	1057	369	1426	35.42
Amino Acid Metabolism	3129	30.63	1255	373	1628	40.44
Non-peptidal Amino Acid Metabolism	468	4.58	182	68	250	6.21
Glycan Biosynthesis and Metabolism	492	4.82	206	72	278	6.91
Polyketides/Nonribosomal Peptides	11	0.11	26	6	32	0.79
Metabolism of Cofactors and Vitamins	644	6.3	325	87	412	10.23

* The modern data represents two healthy adults published by Gill et al. [23] while the ancient data represent results from Z1 and Z2 pooled.

**Table 2:** Comparison of the pathways between modern and ancient data.

Pearson's Chi Square*	P value
KEGG: Z1 to Z2	0.1578
KEGG: Total Z to Modern	<0.00001
KEGG database to Total Z	<0.00001
COG: Z1 to Z2	0.00545
COG database to Total Z	<0.00001

* Pearson's Chi-squared tests were performed in R [39]. The modern data represents two healthy adults published by Gill et al. [23]. KEGG data were calculated from the six pathways on Table 1, while COG data were calculated from the three pathways on Table S3. In the relevant comparisons, we treat KEGG and COG data as expected proportions for samples of equal size to the observed modern or ancient data.

Overall, our ancient phylotype and functional profiles are consistent with those found in living individuals. While we found considerable inter-individual phylotype diversity between the ancient samples, few differences in the functional profiles were observed. Because human microbiome research is still in its pioneering stage, there is a great deal of uncertainty about potential biases of modern microbiome profiles, which equally applies to this ancient study. If our results remain representative in future studies, the evolutionary implications are worth considering.

The classic microbial hypothesis that “everything is everywhere” [30] asserts that microbes are impervious to geographic constraints. The present work lends support to this hypothesis but with the caveat of environment dependent natural selection directing the frequency of microbial distribution in both modern and ancient populations. Yet, our study also raises a question about the “everything is everywhere” hypothesis, specifically, on what is the role of natural selection.

Because of the functional redundancy within microbiotas [29], hypothetically, functional profiles can reach a steady state with a selective pressure while frequencies of bacterial species remain in flux. If this is the case here, then stochastic ecological factors [31] are major contributors to the species observed.

The similarity between the two ancient samples may be attributed to the sampling of a family unit. Alternatively, this similarity may be attributed to a broader biogeographic structure. We raise the hypothesis that human microbiome variation was more geographically structured during pre-Columbian times than that observed today. Because diet is a factor in microbial gut profiles [1], [2], [9] and in pre-Columbian communities, dietary options were more restricted, pre-Columbian resource catchments were more regional than those today, resulting in more restricted exposure to other environs. Additional, population based, studies are required to further test this hypothesis.

As studies begin to define both the modern and ancient human microbiomes we should focus on providing deeper coverage of these communities and consider the effects of important covariates such as diet, sex, geographical and temporal placement, and signatures of disease. Fortunately, as both modern and ancient DNA data can provide valuable dietary information as well as the underlying genetic variation of the host, which includes sex determination [27], we can obtain a broader view of humans as adapted superorganisms and better understand the impact of gut microbiota-target therapies on future health-related research [15].

## Materials and Methods

### Materials

The El Zape samples were originally collected during excavations by Richard and Sheilagh Brooks in the 1960s. Samples were stored at the University of Nebraska State Museum, providing a cool dry place to reduce modern fungal and bacteria growth. Samples were stored in sterile forensic specimen bags. In 2007, samples were sent to the Molecular Anthropology Ancient DNA Laboratory at the University of Oklahoma. This laboratory is a positive pressure clean room, with isolated ventilation. Incoming air passes through ISO 7 (class 10,000) HEPA-filtration. The room is equipped with UVC lighting. Sterile disposable gowns, gloves, hair nets and masks are worn while working in the laboratory.

### DNA extraction methods and contamination controls

Researchers had previously handled the coprolites. To decontaminate, the coprolite surface was brushed with 4% sodium hypochlorite then rinsed with DNA free distilled deionized H_2_0. Approximately 2–5 grams of material were removed from the interior matrix of the coprolite for DNA extraction. As a control for contamination during the procedures, all DNA extractions were performed in tandem with extraction blanks. All DNA extraction and PCR blanks were negative for contamination (Table S4 ).

The following results lend additional support that animal and bacterial contamination was not a confounding factor: 1) the Cueva de los Muertos Chiquitos archaeological site is accessed by hand and toe holds [20] having reduced access by animals 2) the only animal DNA identified from the shotgun reads were human, 3) targeted human mtDNA markers revealed a single, unambiguous, Native American haplogroup per sample, 4) the microbial profile is typical for fecal ecologies, not soil ecologies, and the pattern is consistent with human feces, 5) No modern fecal samples have entered the ancient DNA laboratory and any contamination occurring after sample prep would have been eliminated because of the lack of a MID.

Two DNA extraction protocols were used: 1) the UltraClean™ Mega Soil DNA Isolation Kit (MoBio) for sample Z1, 2) a modified salting out (SO) extraction for sample Z2. MoBio extraction was performed following the manufacturer's specifications. The salting out protocol is preferred for coprolites harboring bone as it includes a bone demineralization step. Importantly, different DNA extraction methods may complicate microbiome studies. For example, gram-positive bacteria are frequently more resistant to cell lysis than gram negative bacteria. Because our results found similar functional profiles between our ancient samples, this confounding factor was not a concern in this study.

### Salting out extraction

The sample was hydrated and lysed using 12 ml of lysis buffer (400 mM NaCl, 10 mM TrisHcl ph 7.5, 2 mM Na_2_EDTA pH8.2) and 800 μl of SDS 20% overnight. 150 μl of Proteinase K at 20 mg/ml (Invitrogen) was added followed by incubation at 65°C for 3 hours. The sample was centrifuged at 4000 RPMs for 10 minutes. The supernatant was transferred to a new 50 ml tube and mixed with 4 ml of 5M NaCl followed by a 10 minute incubation at −20°C. After incubation, the sample was centrifuged at 4000 RPM for 10 minutes. The supernatant was transferred to a new 50 ml tube and mixed with 5 ml of isopropanol and left overnight for precipitation of the nucleic acids. The next day sample was spun at 4000 RPMs for 10 minutes. After removing the supernatant, the sample was washed once with 70% ethanol. The DNA pellet was rehydrated in 4 ml of DNA free distilled deionized H_2_0.

### DNA concentration and inhibitors removal

DNA and blank extractions from both methods, MoBio and SO, were purified and concentrated using Wizard prep SV columns (Promega). 2 ml of each DNA and blank extract were concentrated to a final volume of 50 μl. An additional negative control was used to assess if any of the Wizard prep reagents or columns were contaminated.

### Ancient DNA quantity and quality verification

5 μl of each DNA extract and blank were removed from the ancient lab for DNA quantification prior to and after the library preparation. Four quantification methods were employed to evaluate consistency:

#### Relative method

To infer the DNA concentration and to assess the presence of protein inhibitors, the 260 and 280 nm absorbance was measured using 1μl in a NanoDropTM 1000 Spectrophotometer. This method is only effective for concentrations greater than 5 ng/μl (from laboratory experience) and will not evaluate molecular weights.

#### Indirect method

To infer molecular weights, 1 μl of each sample was mixed with 6× orange loading buffer (NEB) and loaded in a 2% agarose gel using 3 μl of 50bp ladder (Fermentas), 1× SB buffer (728 mM NaOH, 200 mM boric acid) and run at 150 volts for an hour. The gel was stained for 20 minutes in an EtBr bath (1 mg/ml). The results provide a rough estimate of the molecular weight and concentration.

#### Quantitative PCR

Copy number of the human mtDNA, Atopobium spp. 16S rDNA and Enterococcus spp. 16s rDNA were quantified using quantitative PCR (qPCR) using published primers [32]–[34]. The 16s rDNA primers used are specific to species typical of the gut. The standard curves were generated using PCR products from a modern DNA sample. Each qPCR reaction contained the following: 1× High Fidelity PCR Buffer (Invitrogen), 1.5 mM MgSO_3_ (Invitrogen), 333 μM dNTPs (Invitrogen), 170 nM of each primer (IDT), 0.1×SYBR (Molecular Probes), 1 unit of Platinium® Taq high Fidelity (Invitrogen), and 5 μl of DNA template (standard, dilution 1:25 of the extract or water). The temperature profile for the reaction included an initial activation of the enzyme at 94°C for 2 minutes, followed by 60 cycles of the following 94°C for 15 seconds, 54°C (for all pairs of primers) for 15 seconds and 72°C for 15 seconds and a final extension at 72°C for 5 minutes. Melting curve was obtained measuring the fluorescence intensity of the PCR product in a linear denaturation ramp from 55 to 95°C, increasing 0.5°C every 6 seconds. All the qPCR set up was performed in the ancient laboratory in order to avoid external contamination. This method provides accurate estimates of the number of molecules of a specific locus.

#### Agilent 2100 Bioanalyzer

To infer total DNA concentration and molecular weights, 1 μl of each sample was loaded in the Agilent 2100 Bioanalyzer (DNA 7500 Assay). This instrument can quantify a minimum amount of 0.1 nanograms of fragmented DNA of the same size. Results for these methods are provided in Table S4.

To determine if the two coprolites (Z1 and Z2) represented separate individuals, Native American mitochondrial haplogroups were identified by PCR and gel electrophoresis using published methods [32]. The haplogroup results further validate these samples as human.

### Library construction and pyrosequencing

All the steps for library preparation were performed in the ancient laboratory in order to avoid external contamination. While the library preparation protocol requests 5 micrograms of DNA, for sample Z1, we employed published methods for using smaller amounts of DNA [35]. For sample Z1 and Z2, 0.34 mg and 9.29 mg of DNA was used, respectively. Library formation employed Multiplex Identifier Kits (Roche Applied Science). Multiplex Identifier (MID) 1 and 2 were used for samples Z1 and Z2, respectively.

Aliquots of both libraries were removed from the ancient lab to determine the correct adaptor ligation and the number of DNA molecules. The quantity and the quality of each library were assessed using the Agilent 2100 Bioanalyzer. For each library, 2.0 E+07 dilutions were made and then pooled together in a single tube. DNA sample preparation for sequencing on the 454/Roche GS-FLX were performed as described by the manufacturer [36] with modifications to improve the overall yields by replacing the Qiagen MinElute centrifuge columns with an Agencourt AMPure SPRI bead-based purification, removing the manipulations that were recommended to enrich DNA molecules that contain a single A and B adapter ligated on each end of the fragment, to eliminate the steps that resulted in generating a single stranded DNA library [37]. Because ancient DNA molecules represented fragments between 50–800bp, no shearing of the DNA was required. The product from the emulsion PCR was packed in ¼ of a PicoTiterPlate™ and the DNAs were loaded onto a 454/Roche GE-FLX for massively parallel pyrosequencing. The resulting sequence data was assembled using the manufacturer supplied Newbler assembler followed by clustering using Phrap [38].

### Identifying species and function

The FLX run data was exported in FASTA format. These files represent DNA sequences beginning with the MID. A Perl script was used to sort data, identify reads not matching a MID, and use discontiguous BLASTN (blast.ncbi.nlm.nih.gov/Blast.cgi) searches over the whole NCBI refseq_genomic database. Additionally, reads generated from 454 underwent BlastX analysis against KEGG and COG databases using an e-value of 10^−5^ as the threshold.

## Supporting Information

Table S1 The frequency of divisions (phyla) with reads providing greater than 80% identity.
(0.06 MB DOC)

Table S2 Matches putative open reading frames to KEGG pathways.
(0.39 MB DOC)

Table S3 Matches of shotgun sequencing reads to COG pathways.
(0.06 MB DOC)

Table S4 The DNA quantity and quality verification results reflecting finds after 40× concentration.
(0.03 MB DOC)
