# Supplementary material for: Phylotyping and Functional Analysis of Two Ancient Human Microbiomes
Source: PLoS One. 2008 Nov 11;3(11):e3703. doi: 10.1371/journal.pone.0003703 (PMC2577302; doi:10.1371/journal.pone.0003703)
Supplement: Table S1 — The frequency of divisions (phyla) with reads providing greater than 80% identity. (0.06 MB DOC) [file pone.0003703.s001.doc]

**Table S1, Title: The frequency of divisions (phyla) with reads providing greater than 80% identity.**

| **Division** | **Sample Z1** | | **Sample Z2** | |
| --- | --- | --- | --- | --- |
|  | **Counts** | **Percent** | **Counts** | **Percent** |
| Acidobacteria | 27 | 0.54 | 0 | 0.00 |
| Actinobacteria | 380 | 7.65 | 69 | 3.47 |
| Alveolata | 9 | 0.18 | 1 | 0.05 |
| Apicomplexa | 13 | 0.26 | 21 | 1.06 |
| Ascomycetes | 5 | 0.10 | 3 | 0.15 |
| Bacteroidetes | 1025 | 20.63 | 378 | 19.03 |
| candidate division TG1 | 3 | 0.06 | 0 | 0.00 |
| Chlamydiae | 20 | 0.40 | 2 | 0.10 |
| Chlorobi | 43 | 0.87 | 7 | 0.35 |
| Chloroflexi | 65 | 1.31 | 1 | 0.05 |
| Chordata | 93 | 1.87 | 70 | 3.52 |
| Crenarchaeota | 9 | 0.18 | 1 | 0.05 |
| Cyanobacteria | 97 | 1.95 | 15 | 0.76 |
| Deinococcus-Thermus | 40 | 0.81 | 4 | 0.20 |
| Euglenozoa | 11 | 0.22 | 2 | 0.10 |
| Euryarchaeota | 72 | 1.45 | 29 | 1.46 |
| Fibrobacteres | 9 | 0.18 | 0 | 0.00 |
| Firmicutes | 1461 | 29.41 | 1026 | 51.66 |
| Fungi | 48 | 0.97 | 24 | 1.21 |
| Fusobacteria | 2 | 0.04 | 6 | 0.30 |
| Lycopodiophyta | 1 | 0.02 | 0 | 0.00 |
| Metazoa | 22 | 0.44 | 13 | 0.65 |
| Nanoarchaeota | 2 | 0.04 | 0 | 0.00 |
| Planctomycetes | 4 | 0.08 | 0 | 0.00 |
| Proteobacteria | 1366 | 27.50 | 260 | 13.09 |
| Rhodophyta | 3 | 0.06 | 0 | 0.00 |
| Spirochaetes | 43 | 0.87 | 12 | 0.60 |
| Tenericutes | 26 | 0.52 | 18 | 0.91 |
| Thermotogae | 37 | 0.74 | 10 | 0.50 |
| Verrucomicrobia | 15 | 0.30 | 1 | 0.05 |
| Viridiplantae | 17 | 0.34 | 13 | 0.65 |
| **Total** | 4968 | 100.00 | 1986 | 100.00 |
