# Supplementary material for: Phylotyping and Functional Analysis of Two Ancient Human Microbiomes
Source: PLoS One. 2008 Nov 11;3(11):e3703. doi: 10.1371/journal.pone.0003703 (PMC2577302; doi:10.1371/journal.pone.0003703)
Supplement: Table S2 — Matches putative open reading frames to KEGG pathways. (0.39 MB DOC) [file pone.0003703.s002.doc]

Table S2, Title: **Matches putative open reading frames to KEGG pathways.**

|  | **Z1** | **Z2** | **Total Z1+Z2** | **Domains in KEGG** | **freq Z1** | **freq Z2** | **Odds Ratio*** |
| --- | --- | --- | --- | --- | --- | --- | --- |
|
| **1. Metabolism** | **4407** | **1416** | **5823** | **618593** | **0.8405** | **0.8161** | **1.1057** |
| **1.1 Carbohydrate Metabolism** | **1057** | **369** | **1426** | **139571** | **0.2016** | **0.2127** | **1.2001** |
| Glycolysis / Gluconeogenesis | 101 | 59 | 160 | 16043 | 0.0193 | 0.0340 | 1.1714 |
| Citrate cycle (TCA cycle) | 70 | 14 | 84 | 9862 | 0.0134 | 0.0081 | 1.0005 |
| Pentose phosphate pathway | 67 | 26 | 93 | 9960 | 0.0128 | 0.0150 | 1.0968 |
| Pentose and glucuronate interconversions | 41 | 10 | 51 | 4320 | 0.0078 | 0.0058 | 1.3867 |
| Fructose and mannose metabolism | 90 | 31 | 121 | 10524 | 0.0172 | 0.0179 | 1.3505 |
| Galactose metabolism | 85 | 33 | 118 | 5901 | 0.0162 | 0.0190 | 2.3488 |
| Ascorbate and aldarate metabolism | 6 | 1 | 7 | 2645 | 0.0011 | 0.0006 | 0.3109 |
| Starch and sucrose metabolism | 148 | 42 | 190 | 10037 | 0.0282 | 0.0242 | 2.2235 |
| Aminosugars metabolism | 45 | 16 | 61 | 6304 | 0.0086 | 0.0092 | 1.1366 |
| Nucleotide sugars metabolism | 42 | 19 | 61 | 4405 | 0.0080 | 0.0110 | 1.6266 |
| Pyruvate metabolism | 134 | 46 | 180 | 16898 | 0.0256 | 0.0265 | 1.2512 |
| Glyoxylate and dicarboxylate metabolism | 60 | 13 | 73 | 7909 | 0.0114 | 0.0075 | 1.0841 |
| Propanoate metabolism | 64 | 22 | 86 | 13520 | 0.0122 | 0.0127 | 0.7471 |
| Butanoate metabolism | 79 | 28 | 107 | 15182 | 0.0151 | 0.0161 | 0.8278 |
| C5-Branched dibasic acid metabolism | 12 | 3 | 15 | 2276 | 0.0023 | 0.0017 | 0.7741 |
| Inositol metabolism | 2 | 2 | 4 | 1201 | 0.0004 | 0.0012 | 0.3912 |
| Inositol phosphate metabolism | 11 | 4 | 15 | 2584 | 0.0021 | 0.0023 | 0.6818 |
| **1.2 Energy Metabolism** | **359** | **110** | **469** | **58230** | **0.0685** | **0.0634** | **0.9460** |
| Oxidative phosphorylation | 76 | 24 | 100 | 22165 | 0.0145 | 0.0138 | 0.5299 |
| Photosynthesis | 2 | 0 | 2 | 1703 | 0.0004 | 0.0000 | 0.1379 |
| Carbon fixation | 103 | 30 | 133 | 7904 | 0.0196 | 0.0173 | 1.9765 |
| Reductive carboxylate cycle (CO2 fixation) | 55 | 13 | 68 | 7911 | 0.0105 | 0.0075 | 1.0096 |
| Methane metabolism | 13 | 2 | 15 | 4420 | 0.0025 | 0.0012 | 0.3986 |
| Nitrogen metabolism | 82 | 29 | 111 | 9586 | 0.0156 | 0.0167 | 1.3601 |
| Sulfur metabolism | 28 | 12 | 40 | 4541 | 0.0053 | 0.0069 | 1.0347 |
| **1.3 Lipid Metabolism** | **255** | **91** | **346** | **50901** | **0.0486** | **0.0524** | **0.7984** |
| Fatty acid biosynthesis | 44 | 13 | 57 | 7881 | 0.0084 | 0.0075 | 0.8495 |
| Fatty acid metabolism | 20 | 9 | 29 | 10024 | 0.0038 | 0.0052 | 0.3398 |
| Synthesis and degradation of ketone bodies | 0 | 2 | 2 | 1889 | 0.0000 | 0.0012 | 0.1244 |
| Biosynthesis of steroids | 43 | 8 | 51 | 4965 | 0.0082 | 0.0046 | 1.2065 |
| Bile acid biosynthesis | 17 | 9 | 26 | 5655 | 0.0032 | 0.0052 | 0.5400 |
| C21-Steroid hormone metabolism | 1 | 0 | 1 | 156 | 0.0002 | 0.0000 | 0.7529 |
| Androgen and estrogen metabolism | 3 | 5 | 8 | 1494 | 0.0006 | 0.0029 | 0.6290 |
| Glycerolipid metabolism | 54 | 19 | 73 | 6112 | 0.0103 | 0.0110 | 1.4029 |
| Glycerophospholipid metabolism | 33 | 14 | 47 | 8569 | 0.0063 | 0.0081 | 0.6442 |
| Ether lipid metabolism | 3 | 3 | 6 | 933 | 0.0006 | 0.0017 | 0.7554 |
| Sphingolipid metabolism | 29 | 9 | 38 | 1186 | 0.0055 | 0.0052 | 3.7634 |
| Arachidonic acid metabolism | 6 | 0 | 6 | 1500 | 0.0011 | 0.0000 | 0.4698 |
| Linoleic acid metabolism | 2 | 0 | 2 | 537 | 0.0004 | 0.0000 | 0.4375 |
| **1.4 Nucleotide Metabolism** | **510** | **185** | **695** | **51166** | **0.0973** | **0.1066** | **1.5955** |
| Purine metabolism | 268 | 95 | 363 | 29460 | 0.0511 | 0.0548 | 1.4473 |
| Pyrimidine metabolism | 242 | 90 | 332 | 21706 | 0.0462 | 0.0519 | 1.7966 |
| **1.5 Amino Acid Metabolism** | **1255** | **373** | **1628** | **149723** | **0.2394** | **0.2150** | **1.2772** |
| Glutamate metabolism | 151 | 51 | 202 | 12393 | 0.0288 | 0.0294 | 1.9145 |
| Alanine and aspartate metabolism | 155 | 37 | 192 | 12918 | 0.0296 | 0.0213 | 1.7458 |
| Glycine, serine and threonine metabolism | 127 | 46 | 173 | 15604 | 0.0242 | 0.0265 | 1.3023 |
| Methionine metabolism | 61 | 17 | 78 | 7196 | 0.0116 | 0.0098 | 1.2732 |
| Cysteine metabolism | 79 | 26 | 105 | 4593 | 0.0151 | 0.0150 | 2.6852 |
| Valine, leucine and isoleucine degradation | 32 | 12 | 44 | 11796 | 0.0061 | 0.0069 | 0.4381 |
| Valine, leucine and isoleucine biosynthesis | 93 | 32 | 125 | 8994 | 0.0177 | 0.0184 | 1.6325 |
| Lysine biosynthesis | 60 | 42 | 102 | 7363 | 0.0114 | 0.0242 | 1.6272 |
| Lysine degradation | 24 | 10 | 34 | 7209 | 0.0046 | 0.0058 | 0.5540 |
| Arginine and proline metabolism | 95 | 14 | 109 | 7294 | 0.0181 | 0.0081 | 1.7553 |
| Histidine metabolism | 46 | 10 | 56 | 8712 | 0.0088 | 0.0058 | 0.7550 |
| Tyrosine metabolism | 65 | 16 | 81 | 8583 | 0.0124 | 0.0092 | 1.1085 |
| Phenylalanine metabolism | 48 | 7 | 55 | 5289 | 0.0092 | 0.0040 | 1.2214 |
| Tryptophan metabolism | 24 | 13 | 37 | 10381 | 0.0046 | 0.0075 | 0.4186 |
| Phenylalanine, tyrosine and tryptophan biosynthesis | 113 | 23 | 136 | 11563 | 0.0216 | 0.0133 | 1.3815 |
| Urea cycle and metabolism of amino groups | 82 | 17 | 99 | 9835 | 0.0156 | 0.0098 | 1.1824 |
| **1.6 Metabolism of Other Amino Acids** | **182** | **68** | **250** | **28358** | **0.0347** | **0.0392** | **1.0355** |
| beta-Alanine metabolism | 16 | 8 | 24 | 6490 | 0.0031 | 0.0046 | 0.4344 |
| Taurine and hypotaurine metabolism | 25 | 6 | 31 | 2077 | 0.0048 | 0.0035 | 1.7531 |
| Aminophosphonate metabolism | 14 | 4 | 18 | 1183 | 0.0027 | 0.0023 | 1.7872 |
| Selenoamino acid metabolism | 49 | 24 | 73 | 7108 | 0.0093 | 0.0138 | 1.2063 |
| Cyanoamino acid metabolism | 45 | 9 | 54 | 3124 | 0.0086 | 0.0052 | 2.0303 |
| D-Glutamine and D-glutamate metabolism | 5 | 3 | 8 | 1698 | 0.0010 | 0.0017 | 0.5534 |
| D-Alanine metabolism | 12 | 6 | 18 | 1389 | 0.0023 | 0.0035 | 1.5221 |
| Glutathione metabolism | 16 | 8 | 24 | 5289 | 0.0031 | 0.0046 | 0.5330 |
| **1.7 Glycan Biosynthesis and Metabolism** | **206** | **72** | **278** | **20804** | **0.0393** | **0.0415** | **1.5696** |
| High-mannose type N-glycan biosynthesis | 1 | 0 | 1 | 115 | 0.0002 | 0.0000 | 1.0214 |
| N-Glycan degradation | 36 | 5 | 41 | 1190 | 0.0069 | 0.0029 | 4.0469 |
| O-Glycan biosynthesis | 1 | 0 | 1 | 287 | 0.0002 | 0.0000 | 0.4093 |
| Keratan sulfate biosynthesis | 1 | 0 | 1 | 172 | 0.0002 | 0.0000 | 0.6829 |
| Glycosaminoglycan degradation | 25 | 6 | 31 | 1000 | 0.0048 | 0.0035 | 3.6412 |
| Lipopolysaccharide biosynthesis | 12 | 6 | 18 | 4467 | 0.0023 | 0.0035 | 0.4733 |
| Peptidoglycan biosynthesis | 50 | 37 | 87 | 6692 | 0.0095 | 0.0213 | 1.5270 |
| Glycosylphosphatidylinositol(GPI)-anchor biosynthesis | 0 | 1 | 1 | 533 | 0.0000 | 0.0006 | 0.2204 |
| Glycosphingolipid biosynthesis - globoseries | 8 | 3 | 11 | 464 | 0.0015 | 0.0017 | 2.7846 |
| Glycosphingolipid biosynthesis - ganglioseries | 26 | 5 | 31 | 611 | 0.0050 | 0.0029 | 5.9595 |
| Glycan structures - biosynthesis 1 | 1 | 0 | 1 | 1938 | 0.0002 | 0.0000 | 0.0606 |
| Glycan structures - biosynthesis 2 | 7 | 3 | 10 | 1770 | 0.0013 | 0.0017 | 0.6636 |
| Glycan structures - degradation | 38 | 6 | 44 | 1565 | 0.0072 | 0.0035 | 3.3024 |
| **1.8 Biosynthesis of Polyketides and Nonribosomal Peptides** | **26** | **6** | **32** | **1827** | **0.0050** | **0.0035** | **2.0573** |
| Biosynthesis of ansamycins | 1 | 0 | 1 | 111 | 0.0002 | 0.0000 | 1.0582 |
| Polyketide sugar unit biosynthesis | 25 | 6 | 31 | 1716 | 0.0048 | 0.0035 | 2.1219 |
| **1.9 Metabolism of Cofactors and Vitamins** | **325** | **87** | **412** | **59700** | **0.0620** | **0.0501** | **0.8106** |
| Thiamine metabolism | 41 | 8 | 49 | 4800 | 0.0078 | 0.0046 | 1.1991 |
| Riboflavin metabolism | 15 | 4 | 19 | 3659 | 0.0029 | 0.0023 | 0.6099 |
| Vitamin B6 metabolism | 31 | 11 | 42 | 2597 | 0.0059 | 0.0063 | 1.8996 |
| Nicotinate and nicotinamide metabolism | 44 | 15 | 59 | 5857 | 0.0084 | 0.0086 | 1.1832 |
| Pantothenate and CoA biosynthesis | 51 | 10 | 61 | 7109 | 0.0097 | 0.0058 | 1.0079 |
| Biotin metabolism | 12 | 3 | 15 | 2571 | 0.0023 | 0.0017 | 0.6853 |
| Folate biosynthesis | 35 | 4 | 39 | 6368 | 0.0067 | 0.0023 | 0.7194 |
| One carbon pool by folate | 54 | 11 | 65 | 5836 | 0.0103 | 0.0063 | 1.3082 |
| Porphyrin and chlorophyll metabolism | 28 | 14 | 42 | 12140 | 0.0053 | 0.0081 | 0.4064 |
| Ubiquinone biosynthesis | 14 | 7 | 21 | 8763 | 0.0027 | 0.0040 | 0.2815 |
| **1.10 Biosynthesis of Secondary Metabolites** | **108** | **20** | **128** | **16327** | **0.0206** | **0.0115** | **0.9209** |
| Terpenoid biosynthesis | 5 | 4 | 9 | 1489 | 0.0010 | 0.0023 | 0.7100 |
| Limonene and pinene degradation | 20 | 3 | 23 | 6633 | 0.0038 | 0.0017 | 0.4073 |
| Alkaloid biosynthesis I | 10 | 0 | 10 | 759 | 0.0019 | 0.0000 | 1.5475 |
| Alkaloid biosynthesis II | 12 | 4 | 16 | 2166 | 0.0023 | 0.0023 | 0.8677 |
| Penicillin and cephalosporin biosynthesis | 1 | 0 | 1 | 405 | 0.0002 | 0.0000 | 0.2900 |
| Streptomycin biosynthesis | 32 | 6 | 38 | 3521 | 0.0061 | 0.0035 | 1.2677 |
| Novobiocin biosynthesis | 28 | 3 | 31 | 1354 | 0.0053 | 0.0017 | 2.6892 |
| **1.11 Xenobiotics Biodegradation and Metabolism** | **124** | **35** | **159** | **41986** | **0.0237** | **0.0202** | **0.4448** |
| Caprolactam degradation | 6 | 1 | 7 | 3587 | 0.0011 | 0.0006 | 0.2292 |
| Toluene and xylene degradation | 2 | 0 | 2 | 847 | 0.0004 | 0.0000 | 0.2774 |
| gamma-Hexachlorocyclohexane degradation | 6 | 0 | 6 | 2917 | 0.0011 | 0.0000 | 0.2416 |
| 3-Chloroacrylic acid degradation | 1 | 0 | 1 | 2790 | 0.0002 | 0.0000 | 0.0421 |
| 1,1,1-Trichloro-2,2-bis(4-chlorophenyl)ethane (DDT) degradation | 0 | 1 | 1 | 197 | 0.0000 | 0.0006 | 0.5962 |
| 1,2-Dichloroethane degradation | 2 | 0 | 2 | 1066 | 0.0004 | 0.0000 | 0.2204 |
| Tetrachloroethene degradation | 2 | 1 | 3 | 466 | 0.0004 | 0.0006 | 0.7562 |
| Styrene degradation | 4 | 0 | 4 | 1353 | 0.0008 | 0.0000 | 0.3473 |
| 1,4-Dichlorobenzene degradation | 1 | 0 | 1 | 1030 | 0.0002 | 0.0000 | 0.1140 |
| Naphthalene and anthracene degradation | 11 | 4 | 15 | 2598 | 0.0021 | 0.0023 | 0.6782 |
| Ethylbenzene degradation | 11 | 1 | 12 | 1193 | 0.0021 | 0.0006 | 1.1815 |
| Fluorene degradation | 3 | 0 | 3 | 756 | 0.0006 | 0.0000 | 0.4661 |
| Carbazole degradation | 1 | 0 | 1 | 228 | 0.0002 | 0.0000 | 0.5152 |
| Benzoate degradation via CoA ligation | 40 | 13 | 53 | 10000 | 0.0076 | 0.0075 | 0.6225 |
| Benzoate degradation via hydroxylation | 5 | 4 | 9 | 3481 | 0.0010 | 0.0023 | 0.3037 |
| Atrazine degradation | 2 | 0 | 2 | 611 | 0.0004 | 0.0000 | 0.3845 |
| Bisphenol A degradation | 5 | 1 | 6 | 1122 | 0.0010 | 0.0006 | 0.6281 |
| 1- and 2-Methylnaphthalene degradation | 20 | 9 | 29 | 4876 | 0.0038 | 0.0052 | 0.6986 |
| Metabolism of xenobiotics by cytochrome P450 | 2 | 0 | 2 | 2868 | 0.0004 | 0.0000 | 0.0819 |
| **2. Genetic Information Processing** | **410** | **150** | **560** | **79115** | **0.0782** | **0.0865** | **0.8314** |
| **2.1 Transcription** | **240** | **79** | **319** | **17944** | **0.0458** | **0.0455** | **2.0881** |
| Aminoacyl-tRNA biosynthesis | 197 | 68 | 265 | 13418 | 0.0376 | 0.0392 | 2.3198 |
| RNA polymerase | 41 | 11 | 52 | 3527 | 0.0078 | 0.0063 | 1.7317 |
| Basal transcription factors | 2 | 0 | 2 | 999 | 0.0004 | 0.0000 | 0.2352 |
| **2.2 Translation** | **69** | **27** | **96** | **33076** | **0.0132** | **0.0156** | **0.3409** |
| Ribosome | 69 | 27 | 96 | 33076 | 0.0132 | 0.0156 | 0.3409 |
| **2.3 Folding, Sorting and Degradation** | **52** | **20** | **72** | **18794** | **0.0099** | **0.0115** | **0.4500** |
| Protein export | 33 | 15 | 48 | 7886 | 0.0063 | 0.0086 | 0.7149 |
| Type II secretion system | 10 | 3 | 13 | 7428 | 0.0019 | 0.0017 | 0.2056 |
| Type III secretion system | 9 | 2 | 11 | 3480 | 0.0017 | 0.0012 | 0.3713 |
| **2.4 Replication and Repair** | **49** | **24** | **73** | **9301** | **0.0093** | **0.0138** | **0.9219** |
| DNA replication | 49 | 24 | 73 | 9301 | 0.0093 | 0.0138 | 0.9219 |
| **3. Environmental Information Processing** | **376** | **134** | **510** | **89024** | **0.0717** | **0.0772** | **0.6729** |
| **3.1 Membrane Transport** | **299** | **99** | **398** | **56277** | **0.0570** | **0.0571** | **0.8307** |
| ABC transporters - General | 292 | 83 | 375 | 50683 | 0.0557 | 0.0478 | 0.8691 |
| Phosphotransferase system (PTS) | 7 | 16 | 23 | 5594 | 0.0013 | 0.0092 | 0.4829 |
| **3.2 Signal Transduction** | **66** | **32** | **98** | **28333** | **0.0126** | **0.0184** | **0.4063** |
| Two-component system - General | 41 | 17 | 58 | 19873 | 0.0078 | 0.0098 | 0.3428 |
| MAPK signaling pathway | 1 | 5 | 6 | 2435 | 0.0002 | 0.0029 | 0.2894 |
| ErbB signaling pathway | 1 | 0 | 1 | 930 | 0.0002 | 0.0000 | 0.1263 |
| Wnt signaling pathway | 0 | 3 | 3 | 1643 | 0.0000 | 0.0017 | 0.2145 |
| Notch signaling pathway | 0 | 2 | 2 | 525 | 0.0000 | 0.0012 | 0.4475 |
| Jak-STAT signaling pathway | 12 | 3 | 15 | 1433 | 0.0023 | 0.0017 | 1.2295 |
| Phosphatidylinositol signaling system | 11 | 2 | 13 | 1494 | 0.0021 | 0.0012 | 1.0221 |
| **3.2 Signaling Molecules and Interaction** | **11** | **3** | **14** | **4414** | **0.0021** | **0.0017** | **0.3725** |
| Neuroactive ligand-receptor interaction | 0 | 2 | 2 | 1903 | 0.0000 | 0.0012 | 0.1234 |
| Cytokine-cytokine receptor interaction | 11 | 0 | 11 | 1502 | 0.0021 | 0.0000 | 0.8602 |
| Cell adhesion molecules (CAMs) | 0 | 1 | 1 | 1009 | 0.0000 | 0.0006 | 0.1164 |
| **4. Cellular Processes** | **41** | **23** | **64** | **28259** | **0.0078** | **0.0133** | **0.2660** |
| **4.1 Cell Motility** | **18** | **9** | **27** | **17261** | **0.0034** | **0.0052** | **0.1837** |
| Bacterial chemotaxis - General | 5 | 2 | 7 | 7127 | 0.0010 | 0.0012 | 0.1154 |
| Flagellar assembly | 13 | 2 | 15 | 8727 | 0.0025 | 0.0012 | 0.2019 |
| Regulation of actin cytoskeleton | 0 | 5 | 5 | 1407 | 0.0000 | 0.0029 | 0.4174 |
| **4.2 Cell Growth and Death** | **4** | **3** | **7** | **2532** | **0.0008** | **0.0017** | **0.3247** |
| Cell cycle | 1 | 1 | 2 | 1558 | 0.0002 | 0.0006 | 0.1508 |
| Cell cycle - yeast | 3 | 2 | 5 | 974 | 0.0006 | 0.0012 | 0.6030 |
| **4.3 Cell Communication** | **2** | **6** | **8** | **2815** | **0.0004** | **0.0035** | **0.3338** |
| Focal adhesion | 2 | 0 | 2 | 1366 | 0.0004 | 0.0000 | 0.1720 |
| Adherens junction | 0 | 5 | 5 | 528 | 0.0000 | 0.0029 | 1.1123 |
| Tight junction | 0 | 1 | 1 | 921 | 0.0000 | 0.0006 | 0.1275 |
| **4.4 Endocrine System** | **5** | **2** | **7** | **3438** | **0.0010** | **0.0012** | **0.2392** |
| Insulin signaling pathway | 2 | 0 | 2 | 949 | 0.0004 | 0.0000 | 0.2475 |
| Adipocytokine signaling pathway | 1 | 0 | 1 | 522 | 0.0002 | 0.0000 | 0.2250 |
| PPAR signaling pathway | 1 | 1 | 2 | 528 | 0.0002 | 0.0006 | 0.4449 |
| GnRH signaling pathway | 1 | 0 | 1 | 703 | 0.0002 | 0.0000 | 0.1671 |
| Melanogenesis | 0 | 1 | 1 | 736 | 0.0000 | 0.0006 | 0.1596 |
| **4.5 Immune System** | **11** | **1** | **12** | **1911** | **0.0021** | **0.0006** | **0.7376** |
| Hematopoietic cell lineage | 10 | 0 | 10 | 651 | 0.0019 | 0.0000 | 1.8043 |
| Complement and coagulation cascades | 1 | 0 | 1 | 533 | 0.0002 | 0.0000 | 0.2204 |
| Toll-like receptor signaling pathway | 0 | 1 | 1 | 727 | 0.0000 | 0.0006 | 0.1616 |
| **4.6 Development** | **1** | **2** | **3** | **302** | **0.0002** | **0.0012** | **1.1668** |
| Dorso-ventral axis formation | 1 | 2 | 3 | 302 | 0.0002 | 0.0012 | 1.1668 |
| **5. Human Diseases** | **9** | **12** | **21** | **4637** | **0.0017** | **0.0069** | **0.5319** |
| **5.3 Cancers** | **7** | **12** | **19** | **4173** | **0.0013** | **0.0069** | **0.5348** |
| Colorectal cancer | 0 | 1 | 1 | 642 | 0.0000 | 0.0006 | 0.1830 |
| Pancreatic cancer | 1 | 0 | 1 | **542** | 0.0002 | 0.0000 | 0.2167 |
| Glioma | 1 | 0 | 1 | **479** | 0.0002 | 0.0000 | 0.2452 |
| Chronic myeloid leukemia | 1 | 0 | 1 | 560 | 0.0002 | 0.0000 | 0.2097 |
| Basal cell carcinoma | 0 | 1 | 1 | 392 | 0.0000 | 0.0006 | 0.2996 |
| Melanoma | 1 | 5 | 6 | **526** | 0.0002 | 0.0029 | 1.3398 |
| Prostate cancer | 1 | 5 | 6 | 656 | 0.0002 | 0.0029 | 1.0743 |
| Endometrial cancer | 2 | 0 | 2 | **376** | 0.0004 | 0.0000 | 0.6248 |
| **5.1 Neurodegenerative Disorders** | **2** | **0** | **2** | **464** | **0.0004** | **0.0000** | **0.5063** |
| Alzheimer's disease | 1 | 0 | 1 | 226 | 0.0002 | 0.0000 | 0.5197 |
| Huntington's disease | 1 | 0 | 1 | 238 | 0.0002 | 0.0000 | 0.4935 |

* Odds ratios are calculated by the frequency of the category in the total ancient data divided by the frequency of the category in the comparative KEGG dataset.
