# Supplementary material for: Phylotyping and Functional Analysis of Two Ancient Human Microbiomes
Source: PLoS One. 2008 Nov 11;3(11):e3703. doi: 10.1371/journal.pone.0003703 (PMC2577302; doi:10.1371/journal.pone.0003703)
Supplement: Table S3 — Matches of shotgun sequencing reads to COG pathways. (0.06 MB DOC) [file pone.0003703.s003.doc]

**Table S3, Title:** Matches of shotgun sequencing reads to COG pathways.

|  | **Z1** | **Z2** | **Z1+Z2** | **freq Z1** | **freq Z2** | **Domains in COG** | **Odds Ratio*** |
| --- | --- | --- | --- | --- | --- | --- | --- |
| **1. INFORMATION STORAGE AND PROCESSING** | **1582** | **438** | **2020** | **0.23** | **0.26** | **32181** | **1.18** |
| Translation, ribosomal structure and biogenesis | 654 | 174 | 828 | 0.10 | 0.10 | 10572 | 1.47 |
| Transcription | 338 | 93 | 431 | 0.05 | 0.06 | 11271 | 0.72 |
| Replication, recombination and repair | 590 | 171 | 761 | 0.09 | 0.10 | 10338 | 1.38 |
| **2. CELLULAR PROCESSES AND SIGNALING** | **992** | **271** | **1263** | **0.15** | **0.16** | **26172** | **0.91** |
| Cell cycle control, cell division, chromosome partitioning | 72 | 17 | 89 | 0.01 | 0.01 | 1678 | 1.00 |
| Signal transduction mechanisms | 177 | 44 | 221 | 0.03 | 0.03 | 7683 | 0.54 |
| Cell wall/membrane/envelope biogenesis | 268 | 82 | 350 | 0.04 | 0.05 | 7858 | 0.84 |
| Cell motility | 175 | 49 | 224 | 0.03 | 0.03 | 2747 | 1.53 |
| Posttranslational modification, protein turnover, chaperones | 300 | 79 | 379 | 0.04 | 0.05 | 6206 | 1.15 |
| **3. METABOLISM** | **3423** | **782** | **4205** | **0.50** | **0.47** | **64577** | **1.22** |
| Energy production and conversion | 535 | 90 | 625 | 0.08 | 0.05 | 9830 | 1.19 |
| Carbohydrate transport and metabolism | 664 | 170 | 834 | 0.10 | 0.10 | 10816 | 1.45 |
| Amino acid transport and metabolism | 950 | 229 | 1179 | 0.14 | 0.14 | 14939 | 1.48 |
| Nucleotide transport and metabolism | 329 | 90 | 419 | 0.05 | 0.05 | 3922 | 2.01 |
| Coenzyme transport and metabolism | 322 | 66 | 388 | 0.05 | 0.04 | 6582 | 1.11 |
| Lipid transport and metabolism | 128 | 36 | 164 | 0.02 | 0.02 | 5201 | 0.59 |
| Inorganic ion transport and metabolism | 280 | 65 | 345 | 0.04 | 0.04 | 9232 | 0.70 |
| Secondary metabolites biosynthesis, transport and catabolism | 215 | 36 | 251 | 0.03 | 0.02 | 4055 | 1.16 |
| **4. POORLY CHARACTERIZED** | **833** | **169** | **1002** | **0.12** | **0.10** | **36604** | **0.51** |
| General function prediction only | 665 | 134 | 799 | 0.10 | 0.08 | 22721 | 0.66 |
| Function unknown | 168 | 35 | 203 | 0.02 | 0.02 | 13883 | 0.27 |

* Odds ratios are calculated by the frequency of the category in the total ancient data divided by the frequency of the category in the comparative COG dataset.
