# Supplementary material for: Phylotyping and Functional Analysis of Two Ancient Human Microbiomes
Source: PLoS One. 2008 Nov 11;3(11):e3703. doi: 10.1371/journal.pone.0003703 (PMC2577302; doi:10.1371/journal.pone.0003703)
Supplement: Table S4 — The DNA quantity and quality verification results reflecting finds after 40× concentration. (0.03 MB DOC) [file pone.0003703.s004.doc]

Table S4, Title: **The DNA quantity and quality verification results reflecting finds after 40X concentration.**

|  | **Relative** | | **Indirect** | **Quantitative** | | | **Agilent 2100 Bioanalyzer (DNA 7500 Assay)** | |
| --- | --- | --- | --- | --- | --- | --- | --- | --- |
|  | ng/ul | 260/280 |  | mtDNA | Atopobium | Enterococcus | Average size bp | ng/ul |
| Z1 DNA Extraction* | 71 | 1.74 | DNA smearing | Positive | Positive | Positive | 50 to 852 | 14.1 |
| Z1 Extraction Blank * | -7.7 | 2.12 | None | None | None | None | None | None |
| Z2 DNA Extraction ** | 1817.1 | 1.9 | DNA smearing | Positive | Positive | Positive | 50 to 200 | 387 |
| Z2 Extraction Blank ** | -11.92 | 2.4 | None | None | None | None | None | None |
| Wizard prep SV Blank | -7.87 | 1.96 | None | None | None | None | None | None |

* Z1 was extracted using the MoBio protocol

* Z2 was extracted using the Silica method
